# Effect of fatigue on intermuscular EMG-EMG coupling during bench press exercise at 60% 1RM workload in males

**DOI:** 10.3389/fnhum.2024.1472075

**Published:** 2024-10-22

**Authors:** Lejun Wang, Haifeng Tao, Qing Chen, Minjie Qiao, Xiaoqian Song, Wenxin Niu

**Affiliations:** ^1^Sport and Health Research Center, Shanghai YangZhi Rehabilitation Hospital (Shanghai Sunshine Rehabilitation Center), Physical Education Department, Tongji University, Shanghai, China; ^2^School of Medicine, Tongji University, Shanghai, China

**Keywords:** bench press, muscle fatigue, EMG, intermuscular coupling, phase synchronization analysis

## Abstract

**Objective:**

To explore the neuromuscular control mechanism and quantifying the fatigue response during bench press exercise is important references to prescribe an appropriate exercise program. However, current literature struggles to provide a concrete conclusion on the changes of intermuscular EMG-EMG coupling between synergistic and antagonist muscles during the exercise. Thus, the current study was designed to reveal fatigue-related changes of intermuscular EMG-EMG coupling during bench press exercise.

**Methods:**

Thirty-one healthy male participants performed a bench press exercise on the Smith machine at 60% One Repetition Maximum (1RM) workload to exhaustion, while surface electromyographic signals (sEMG) were collected from triceps brachii (TB), biceps brachii (BB), anterior deltoid (AD), posterior deltoid (PD), and pectoralis major (PM). Surface EMG signals were divided into the first half and second half of the bench press exercise. Phase synchronization index (PSI) was calculated between sEMG of synergistic muscle pairs AD-TB, AD-PM and antagonist muscle pairs BB-TB, AD-PD.

**Results:**

EMG power of TB, AD, PD, PM muscles in alpha (8–12 Hz) frequency band and EMG power of each muscle in beta (15–35 Hz), and gamma (35–60 Hz) frequency bands were all increased during the second half of contraction compared with the first half of contraction. PSI of gamma frequency band was significantly decreased in BB-TB muscle pair while EMG-EMG coupling of AD-TB in gamma frequency band was significantly increased during the second half of contraction compared to the first half of contraction.

**Conclusions:**

The results indicated a decrease of interconnection between synchronized cortical neurons and the motoneuron pool of BB and TB, and an increase of interconnection between AD-TB muscles during fatiguing bench press exercise at 60% 1RM workload. The changes of intermuscular coupling may be related to the supraspinal modulations to compensate for the decrease of muscle force as well as a result of unbalanced changes of agonist and antagonist muscle contractility.

## 1 Introduction

Bench press is a classic type of exercise which is commonly accompanied with measuring or training upper-body strength (Schick et al., 2010; Golas et al., 2017). During exercise and training, bench press are often carried out for a number of sets at submaximal loads of a certain percentage of 1-RM to exhaustion, which may induce severe muscle fatigue of upper extremity (van den Tillaar and Saeterbakken, 2013; Tsoukos et al., 2021). As a commonly occurred phenomenon during training, muscle fatigue has been suggested to be a necessary stimulus for training gains while excessive local muscle fatigue may also lead to potential musculoskeletal disorder and injury risks (Schott et al., 1995; Delvaux et al., 2017). To explore the neuromuscular control mechanism and quantifying the fatigue response during bench press exercise is important references to prescribe an appropriate exercise program (Byrne et al., 2004; Carroll et al., 2017; Wang et al., 2021).

Muscle fatigue is an exercise-induced reduction in the muscle's maximal capacity to generate force or power output (Vøllestad, 1997). It represents a complex phenomenon encompassing both peripheral and central mechanisms (Danna-Dos et al., 2010). Muscle fatigue may lead to an increase in muscle force fluctuations, alternating levels of single muscle activity as well as changes in the muscle coordination patterns as a result of descending drive adjustment. Electroencephalographic (EEG)—electromyography (EMG) synchronization analysis is an effective approach to explore the central modulation information of co-contracted muscles (Boonstra and Breakspear, 2012; Farina et al., 2014). In previous researches, the synchronization of co-contracted muscles oscillations has been mainly evaluated in frequency (coherence analysis) and phase (phase synchronization analysis) domain (van Asseldonk et al., 2014; Pizzamiglio et al., 2017; Wang et al., 2015). In particular, EEG and EMG signals have been revealed to be phase locked (Mima et al., 2000; Ushiyama et al., 2011), which demonstrates that phase synchronization activities of EMG signals between co-contracted muscles may reflect cortical related modulation information (Wang et al., 2015).

In exercise-induced muscle fatigue related intermuscular EMG-EMG coupling changes, an enhancement of EMG-EMG coupling between synergistic and antagonist muscles has been observed during sustained isometric contractions and dynamic motor tasks for both healthy participants as well as patients, which may indicate an increased intermuscular common neural inputs as a result of muscle fatigue (Kattla and Lowery, 2010; Charissou et al., 2016, 2017). However, fatigue-related decrease of intermuscular coupling has also been found in previous researches (Dos Santos et al., 2020). Up to now, current literature struggles to provide a concrete conclusion on the changes of intermuscular EMG-EMG coupling between synergistic and antagonist muscles during bench press exercise.

The aim of this study was to investigate fatigue-related changes of intermuscular EMG-EMG coupling of both synergistic and antagonistic muscles during bench press exercise. Surface EMG signals of agonist muscles triceps brachii (TB), anterior deltoid (AD), pectoralis major (PM) and antagonist muscles biceps brachii (BB), posterior deltoid (PD) were recorded during bench press of 60% 1RM workload till exhaustion. EMG-EMG coupling of antagonistic muscles were compared between the first half (stage 1 with minimal fatigue) and second half (stage 2 with severe fatigue) of the exercise.

## 2 Materials and methods

### 2.1 Subjects

Thirty-one young man volunteers (age 19.39 ± 2.01 years, height 174.86 ± 4.86 cm and weight 63.80 ± 7.44 kg) participated in this study. The subjects were all healthy with no known neuromuscular disorders or musculoskeletal injuries of the neuromuscular system. Participants were excluded from the study if they had musculoskeletal pain, injury, illness that might reduce maximal effort or experienced pain during testing. The participants were instructed to refrain from any additional resistance training targeting the upper body during the 72 h before testing to avoid fatigue. Each participant was informed of the test protocol and was volunteered to participate the experiment. The experiment was approved by the Ethics Committee of Tongji University (No. 2020TJDX006).

### 2.2 Data recording

#### 2.2.1 Experimental setup

The experiment was conducted in laboratory with the indoor temperature of about 24°C. The formal experiment comprised of two sessions. During the first testing session, subjects performed a One Repetition Maximum (1RM) bench press test to determine the workload of 1RM for each participant. The second testing session occurred at least 7 days after the 1RM bench press test. During the second session, participants performed a fatiguing bench press exercise at 60% 1RM workload on a Smith Machine (Matrix Fitness, Johnson Health Tech, Cottage Grove, MN, USA).

During the fatiguing bench press test, participants started the exercise lying in the supine position on the bench and grasped the barbell at a comfortable width. The elbows performed flexion comfortably within the coronal, and the wrists were kept in a neutral position. Upon verbal command, subjects concentrically pushed the barbell until executing full elbow Extension and then immediately eccentrically lowered the barbell until the chest was touched, ~3 cm superior to the xiphoid process (Orange et al., 2019). To maximize external validity, lifting cadence was determined by the tempo that each subject felt was most natural to him (Schick et al., 2010), with a 2-s rest between the two successive repetitions. During the whole exercise, the participant's head and back (thoracic area) were kept in contact with the bench to prevent any inertial motion. The participants were encouraged to perform the bench press at a certain pace of 4.096 s for one bench press trial for as many repetitions as possible till they cannot complete the motor task at the certain rhythm.

**Figure 1:**
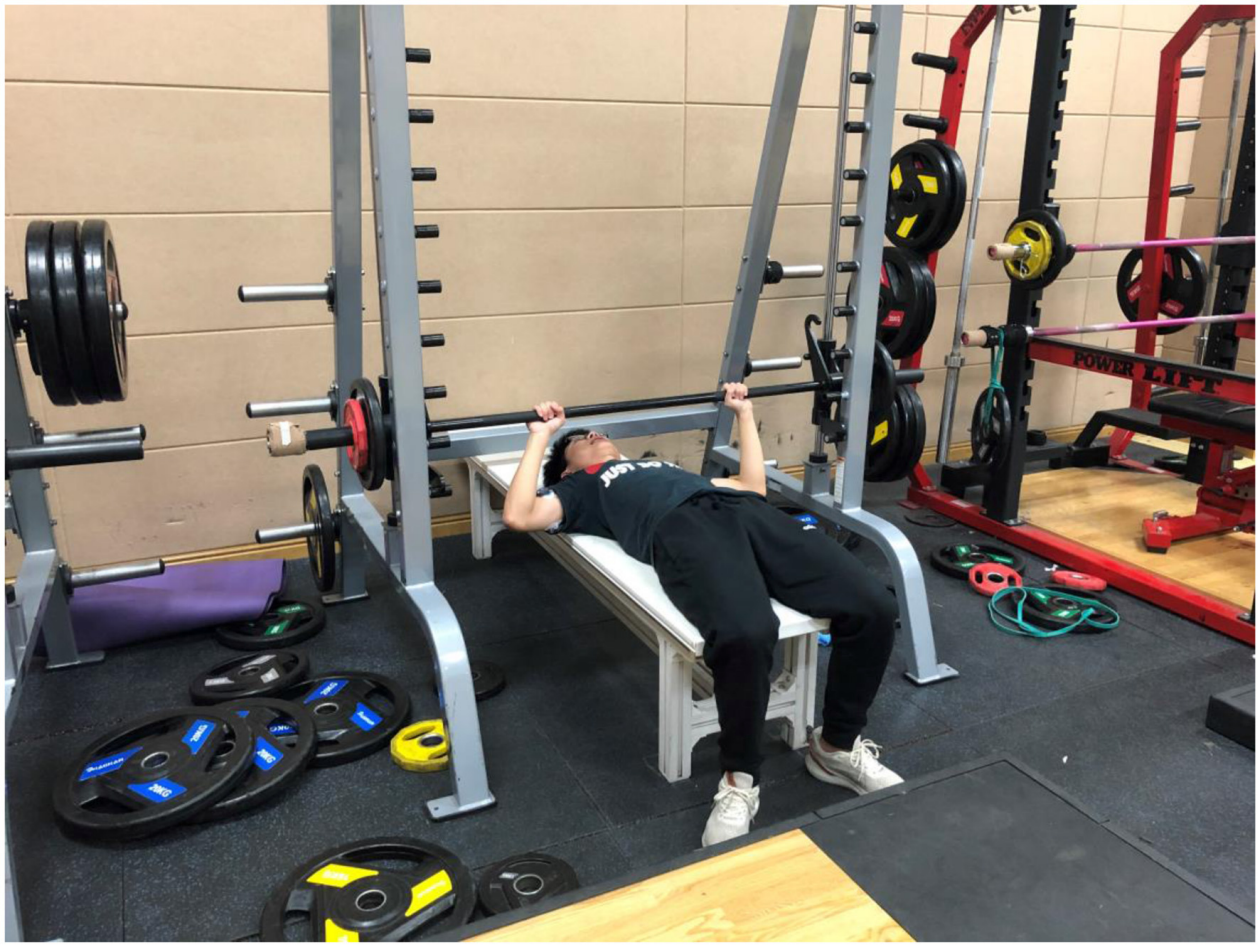
Example diagram of fatiguing bench press exercise. All participants started the exercise lying in the supine position on the bench. During the whole exercise, the participant's head and back (thoracic area) were kept in contact with the bench to prevent any inertial motion. The participants were encouraged to perform the bench press as many repetitions as possible till exhaustion.

During the fatiguing bench press exercise, triaxial acceleration data of barbell were sampled at 100 Hz using a triaxial accelerometric sensor (Kinv TS) fixed to the right edge of the barbell. To ensure accurate results, the X-, Y-, and Z-axes were consistently perpendicular to the sagittal, coronal, and horizontal planes, respectively. Besides, surface EMG signals of right triceps brachii (TB), biceps brachii (BB), anterior deltoid (AD), posterior deltoid (PD), and pectoralis major (PM) muscles were recorded. The example diagram of fatiguing bench press exercise has been showed as Figure 1.

#### 2.2.2 1RM test

Subjects performed a 1RM bench press test with a standard barbell and typical load according to the National Strength and Conditioning Association's guidelines for maximal strength testing. A self-selected load bench press warm-up was first performed, the workload of which allowed each subject to finish 6–10 repetitions (~50% predicted 1 RM) of the exercise. Then, after 1–5 min of rest, subjects then select a weight based on the previous effort which allows them to perform three repetitions (~80% predicted 1 RM). Subjects then progressively increased resistance by 5–10% of the previous attempt. The 1RM was determined within five attempts, and a 3–5-min rest period was provided between each attempt.

#### 2.2.3 EMG measurement

Surface EMG was recorded from the long head of right TB, BB, AD, PD, and PM using a wireless EMG system (BTS FREEEMG 1000, BTS, Garbagnate Milanese MI, Italy) with a 2-cm inter-electrode distance. Specifically, the electrodes were placed as follows: on the TB long head at 50% on the line between the posterior crista of the acromion and the olecranon at two finger widths medial to the line, on the BB at the line between the medial acromion and the fossa cubit at 1/3 from the fossa cubit, on the AD at 1.5 cm distal and anterior to the acromion, on the PD at the area about two fingerbreadths behind the angle of the acromion, and on the PM upper portion at the midclavicular line over the second intercostal space (Rodriguez-Ridao et al., 2020). Prior to positioning the electrodes, the skin was shaved, lightly abraded with fine emery paper and cleaned with alcohol to obtain low inter-electrode resistance. Signals were converted from analog to digital with a sampling rate of 1,000 Hz (Rodriguez-Ridao et al., 2020).

### 2.3 Data processing and analysis

#### 2.3.1 EMG amplitude, median frequency, and power analysis

Raw EMG signals recorded from BB, TB, AD, PD, and PM muscles were inspected and signals recorded during the rest time between each two successive repetitions were discarded. EMG signals were then band-pass filtered at 5–500 Hz offline using a 4th order zero-phase-shift Butterworth filter. The EMG median frequency (MF) and power spectrum were acquired based on Fourier transform of the filtered EMG signals. The filtered EMG signals were full wave rectified. Following full wave rectification, the EMG signals were root mean squared with a 100 ms moving rectangular window to create a linear envelope. EMG root mean square (RMS) of each muscle was calculated based on the EMG envelope. EMG power, MF and RMS were calculated for each non-overlapping 4.096-s epochs during the fatiguing bench press exercise. MF and RMS were time normalized to 100% contraction duration time, while EMG power in the alpha (8–12 Hz), beta (15–35 Hz), and gamma (35–60 Hz) frequency bands were averaged for each epoch during the first and second halves of the fatiguing bench press exercise.

#### 2.3.2 Phase synchronization analysis

EMG signals was firstly divided into two equal-length segments: EMG of the first half and second half of the fatiguing bench press exercise. The EMG of the first and second half were filtered for the frequency ranges 8–12 Hz (alpha band), 15–35 Hz (beta band), and 35–60 Hz (gamma band) using a 4th order zero-phase-shift Butterworth filter. Phase synchronization index (PSI) in alpha, beta, and gamma frequency bands between EMG of synergistic (AD-TB and AD-PM) and antagonist (BB-TB and AD-PD) muscle pairs were calculated as:


(1) $$\begin{array}{l} PSI=\sqrt{{\langle cos\;\theta_{XY}^{H}\left(t\right)\rangle }_{t}^{2}+{\langle sin\;\theta_{XY}^{H}\left(t\right)\rangle }_{t}^{2}} \end{array}$$

Where 〈.〉_*t*_ means the average of all the values and


(2) $$\begin{array}{l} \theta_{XY}^{H}\left(t\right)=n\theta_{X}^{H}\left(t\right)-m\theta_{Y}^{H}\left(t\right) \end{array}$$

In which $\theta_{X}^{H}\left(t\right)$ is the phase angle calculated based on the Hilbert transformation of the EMG signals. In all cases, m and n were assigned a value of 1 according to previous study (Quian et al., 2002). PSI was devised to quantify the phase synchronization between two oscillators of different frequencies. PSI = 0 indicated independent phases, i.e., a complete lack of interaction, and PSI = 1 indicated perfect interaction (Rosenblum et al., 2006).

Data processing was performed using MATLAB 2017Ra software (The MathWorks Inc., Natick, MA, USA).

### 2.4 Statistical analysis

Statistical analysis was performed using SPSS 19.0 for windows (SPSS, Inc., Chicago, IL, USA). Normality was tested by means of the Kolmogorov–Smirnov test. A repeated-measures analysis of variance was used to determine the significance of EMG RMS, MF in different contraction periods. Pearson cross-correlation analysis was used to observe the correlation between EMG indices (RMS and MF) and contraction duration time (20, 40, 60, 80, and 100% of contraction duration time). The difference of average EMG power and phase synchronization index in the alpha (8–12 Hz), beta (15–35 Hz), and gamma (35–60 Hz) frequency bands during first and second half contraction were tested using paired sample *t*-test. All significance thresholds were fixed at α = 0.05.

## 3 Results

**Figure 2:**
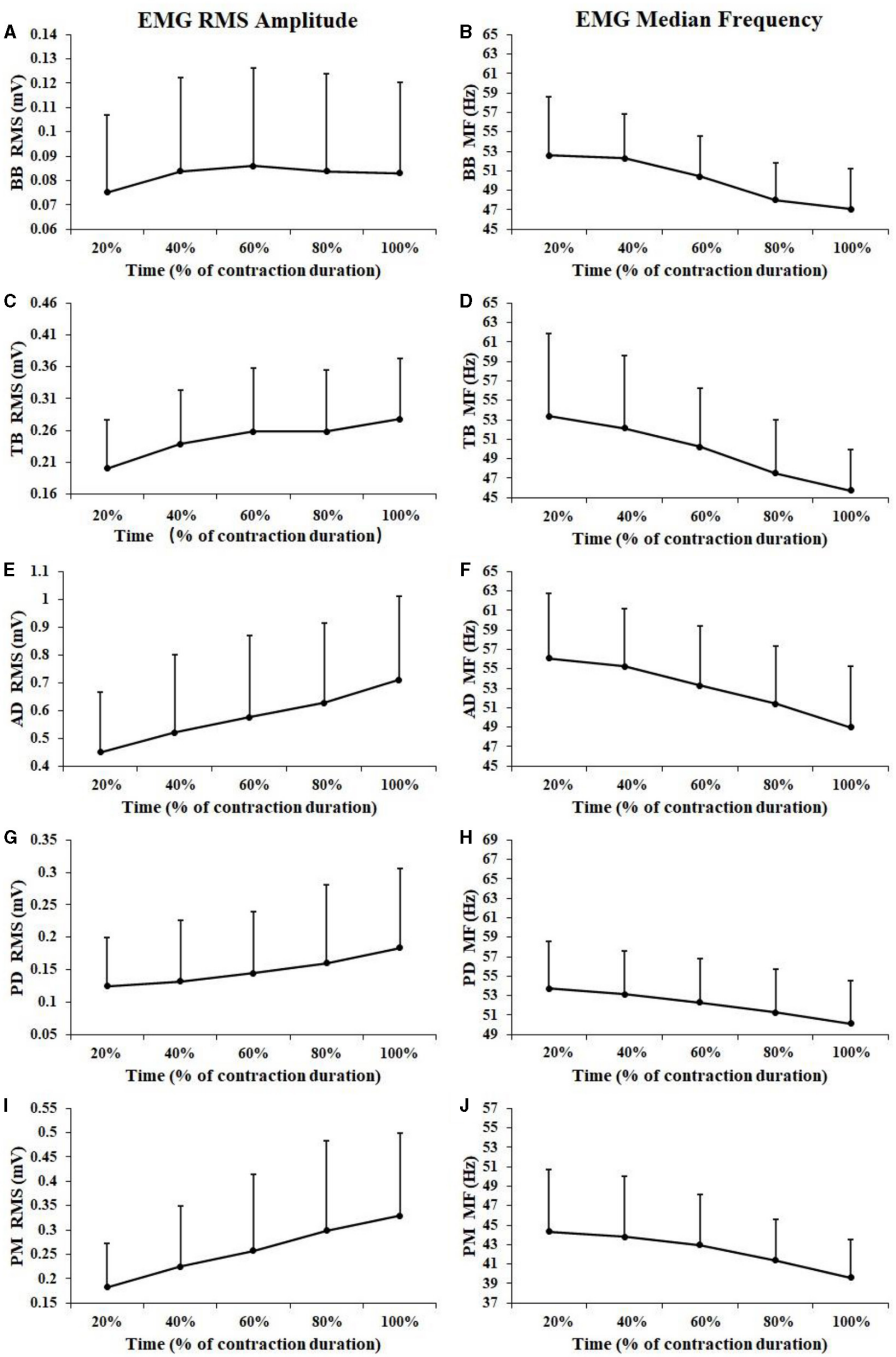
EMG RMS and MF changes of all subjects during the sustained fatiguing contraction of the BB **(A, B)**, TB **(C, D)**, AD **(E, F)**, PD **(G, H)**, and PM **(I, J)** muscle. For each subject, data of EMG RMS and MF has been normalized relative to the values at the beginning of the contraction and the time base has been normalized to 100%.

**Figure 3:**
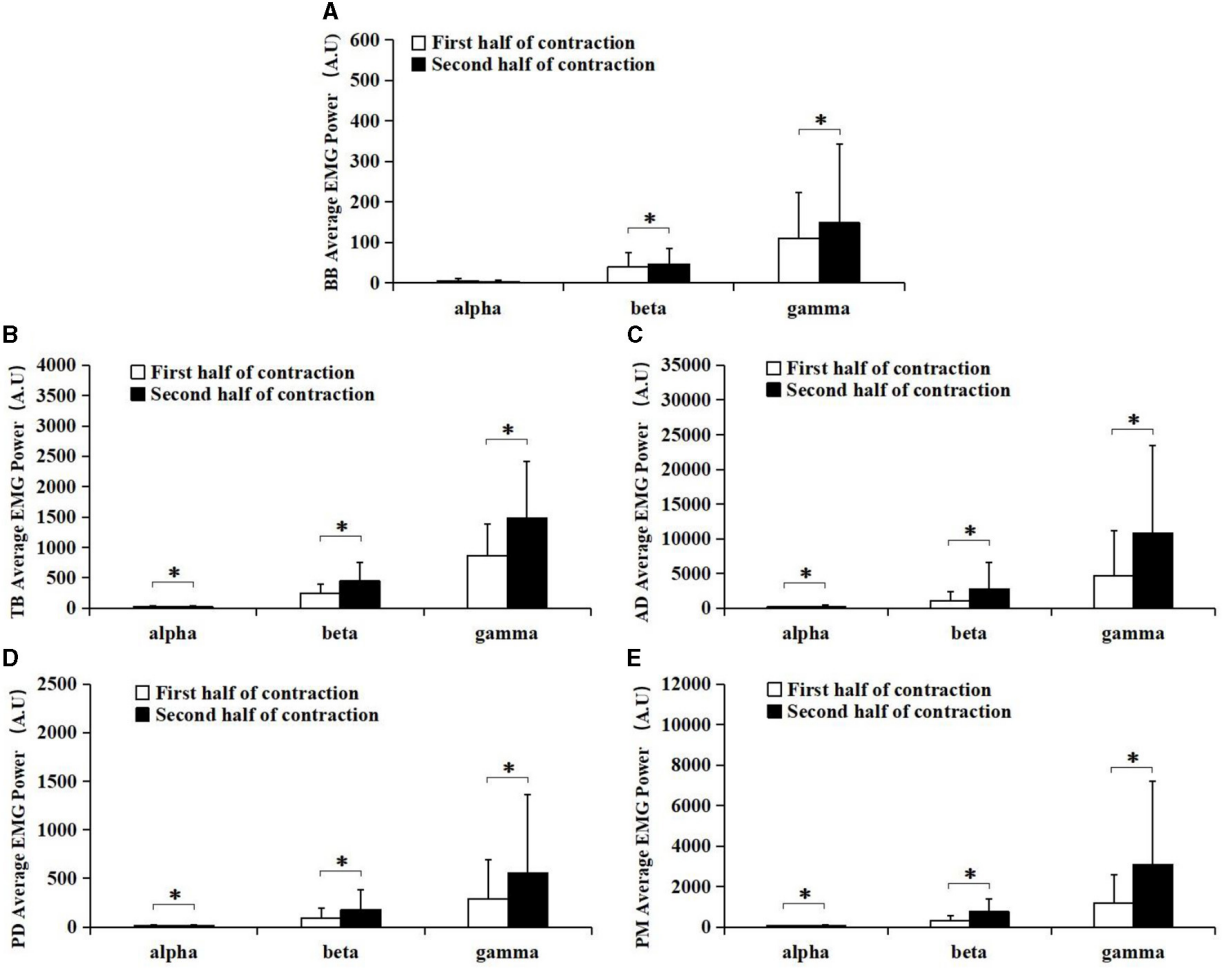
Comparisons of the average EMG power of BB **(A)**, TB **(B)**, AD **(C)**, PD **(D)**, and PM **(E)** muscles at alpha (8–12 Hz), beta (15–35 Hz), and gamma (35–60 Hz) frequency bands between the first (white bar) and second (black bar) half of the contraction. Except for the average EMG power of BB muscle in the alpha band, all muscles in the alpha, beta, and gamma band were significantly higher during the second half of contraction compared to the first half of contraction. *Demonstrated a significant difference of observed index between the first and the second half of contraction.

In this study, the workload of 1RM and 60% 1RM were 43.79 ± 5.58 and 26.27 ± 3.35 kg, respectively. Figure 2 showed EMG RMS and MF changes of all subjects during the sustained fatiguing contraction. The number of repetitions for the fatiguing bench press exercise was 15.35 ± 5.34. As illustrated in Figure 3, during the fatiguing contraction EMG RMS of TB, AD, PD, BB, and PM all increased and EMG MF decreased progressively during the fatiguing bench press exercise. A repeated-measures analysis of variance results revealed that EMG RMS of BB, TB, AD, PD, and PM muscles all showed a significant difference among five contraction periods (BB: *F* = 7.635, *P* < 0.001; TB: *F* = 22.566, *P* < 0.001; AD: *F* = 52.440, *P* < 0.001; PD: *F* = 11.536, *P* < 0.001; PM: *F* = 31.671, *P* < 0.001), as well as EMG MF (BB: *F* = 32.258, *P* < 0.001; TB: *F* = 30.061, *P* < 0.001; AD: *F* = 52.262, *P* < 0.001; PD: *F* = 14.035, *P* < 0.001; PM: *F* = 31.959, *P* < 0.001). Besides, a significant positive correlation between EMG RMS and contraction duration time was observed in all muscles except for BB muscle (BB: *r* = 0.065, *P* = 0.424; TB: *r* = 0.265, *P* = 0.001; AD: *r* = 0.309, *P* < 0.001; PD: *r* = 0.202, *P* = 0.012; PM: *r* = 0.333, *P* < 0.001), while significant negative correlations between EMG MF and contraction duration time were observed for all the five tested muscles (BB: *r* = −0.425, *P* < 0.001; TB: *r* = −0.403, *P* < 0.001; AD: *r* = −0.384, *P* < 0.001; PD: *r* = −0.275, *P* = 0.001; PM: *r* = −0.305, *P* < 0.001).

The average EMG power of BB, TB, AD, PD, and PM in alpha (8–12 Hz), beta (15–35 Hz), and gamma (35–60 Hz) frequency bands during the first and second half of contraction were presented in Figure 3. It can be observed from the figure that EMG power of TB, AD, PD, PM muscles in alpha (8–12 Hz) frequency band and EMG power of each muscle in beta (15–35 Hz), and gamma (35–60 Hz) frequency bands were all increased during the second half of contraction (black bar) compared with the first half of contraction (white bar).

**Figure 4:**
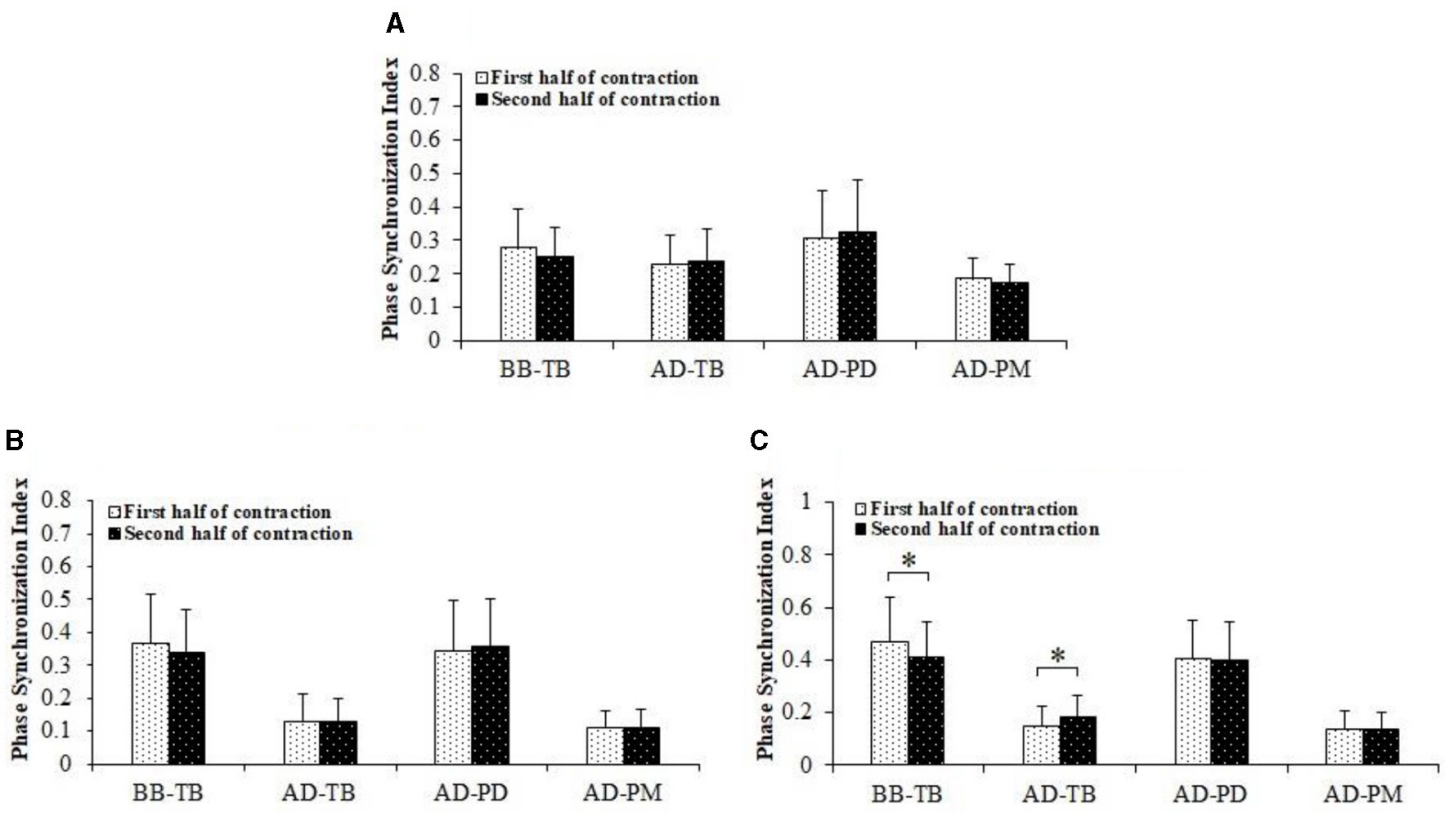
Comparisons of PSI between the first and second half of the fatiguing bench press exercise. The data have been averaged over all subjects. The PSI of BB-TB in the gamma band was significantly decreased while PSI of AD-TB was significantly increased in gamma frequency band during the second half of contraction compared to the first half of contraction. **(A)** Alpha band. **(B)** Beta band. **(C)** Gamma band. *Demonstrated a significant difference of observed index between the first and the second half of contraction.

Figure 4 displayed comparisons of PSI between the first and second half of the fatiguing contraction. There was a significant decrease of PSI in the gamma frequency band for BB-TB muscle pair during the second half of the contraction compared to the first half contraction (*P* = 0.006). A significant increase of PSI in the gamma frequency band for AD-TB muscle pair was also found during the second half of contraction compared to the first half contraction (*P* = 0.013). No significant difference of PSI was found between the first and second half of contraction in other frequency bands for BB-TB and AD-TB muscle pair, and in alpha, beta and gamma frequency bands for the other two muscle pairs.

## 4 Discussion

The aim of this study was to investigate fatigue-related changes of EMG-EMG coupling between synergistic and antagonistic muscles during bench press exercise. It was found that PSI of gamma frequency band was significantly decreased in BB-TB muscle pair while EMG-EMG coupling of AD-TB in gamma frequency band was significantly increased during the second half of contraction compared to the first half of contraction. As far as we know, it is the first study to explore the effect of muscle fatigue on intermuscular coupling during bench press exercise.

During the fatiguing contraction of bench press exercise, EMG RMS of both agonist muscle (TB, AD, and PM) and antagonist muscles (BB and PD) increased progressively with the increase of movement repetitions, while EMG median frequency showed significantly decrease tendency during the fatiguing task. The increase of EMG RMS and decrease of EMG MF have been suggested to be classical characteristics of muscle fatigue induced by exercise. The enhancement of EMG RMS may suggest the increase of motor unit recruit number as well as motor unit firing rates as a result of muscle fatigue, while the decrease of EMG MF may also demonstrate series of changes occurred in peripheral and central sites induced by muscle fatigue (Cifrek et al., 2009; Enoka et al., 2011). Therefore, the results demonstrated that the fatigue of tested muscles developed progressively as the increase of exercise time and repetition numbers during the fatiguing bench press test.

In previous researches concerning fatigue-related changes of intermuscular coupling, an enhancement of EMG-EMG oscillatory activities between synergistic and antagonist muscles has been mainly observed during sustained isometric contractions and dynamic motor tasks for both healthy participants as well as neuromuscular impaired patients (Charissou et al., 2017; Walker et al., 2018; Dos Santos et al., 2020). However, decreases of intermuscular coupling have also been found as a result of muscle fatigue in previous researches (Millet and Lepers, 2004; Padua et al., 2006). Up to now, current literature struggles to provide a concrete conclusion on the changes of intermuscular EMG-EMG coupling between synergistic and antagonist muscles during bench press exercise.

In this study, PSI in gamma frequency band for antagonist muscle pair BB-TB was significantly decreased while PSI of synergistic muscle pair AD-TB was significantly increased during the second half of contraction compared to the first half of contraction, which may indicate a weakening of EMG-EMG coupling between BB-TB and an enhancement of coupling between AD-TB muscle pairs as a result of muscle fatigue. In previous researches, PSI in gamma frequency band has been suggested to be closely related to the common neural inputs of the co-contracted muscles in strong isometric and dynamic voluntary contractions (Gwin and Ferris, 2012; Ushiyama et al., 2012). Therefore, the lower PSI found in BB-TB may indicate a decreased descending common drive of antagonist muscle pair BB-TB and an increased common drive of synergistic muscle pair AD-TB as a result of muscle fatigue during bench press exercise. As both agonist and antagonist muscles performed dynamic contractions during bench press exercise in the current research, central control activities may be predominant related to the dynamic contractions of upper limb muscles, thus the significant changes of EMG-EMG coupling in gamma frequency band can be explained.

During bench press exercise, it has been revealed that EMG activity of TB and AD was found to be higher than other muscles, indicating a significant role of TB and AD as prime mover during the bench press exercise, and thus may be more fatigued and would receive more central modulations during the fatiguing bench process exercise (Moras et al., 2010; van den Tillaar and Saeterbakken, 2014; Stronska et al., 2022). The fatigue would increase the number of recruited motor units and more fast fibers of AD and TB would be recruited (Walker et al., 2018; Griffin et al., 1998). It has been suggested that synchronization of motoneuron inputs may help to overcome reduced excitability of the motoneuron pool during fatigue (Kattla and Lowery, 2010). Synchronized inputs are more effective at recruiting neurons than asynchronous inputs (Murthy and Fetz, 1994; Shen et al., 2024). As a result, muscle fatigue would induce a more synchronized control strategy of AD and TB muscles and thus increase the PSI of AD-TB muscle pair. Therefore, the increase of intermuscular coupling between synergistic muscle pair AD-TB may be related to the supraspinal modulations to compensate for the decrease of muscle contractility as a result of fatigue.

On the other hand, BB has been suggested to act as one of the most important stabilizer and antagonist muscle during bench press exercise, while owing to the lower intensity contraction and increased stability offered by the Smith machine, the stabilizing role of AD and PD may be weakened in the current research (Schick et al., 2010; Saeterbakken et al., 2011, 2016). However, the antagonist muscles of BB would not fatigue during the fatiguing contraction. Specifically, EMG amplitude of BB muscle showed no significant changes during the fatiguing contraction, which may indicate insignificant changes of motor unit number and motor unit firing rate during the second half of contraction compared to the first half. Therefore, the desynchronous changes of BB and TB would result in the decrease of PSI between BB-TB muscle pair (Wang et al., 2015). So the decline of intermuscular coupling between antagonist muscle pair BB-TB may be explained as a result of unbalanced changes of muscle contractility induced by bench press exercise.

However, limitations should also be acknowledged in the current research. First, in order to maximize external validity, subjects performed bench press exercises at their natural pace, which may induce different speeds of movement among subjects and influence muscle activation in the current study. Second, data were only collected and analyzed from the upper limbs of the right side, and the influence of imbalance performance between the left and right sides has not been considered. Thirdly, in this study, we only collected five main muscles according to previous research studies, and other muscles, such as the middle deltoid, upper trapezius, and latissimus dorsi, have not been tested and analyzed (Dunnick et al., 2015). Lastly, the influence of cross-talk has raised concern in previous researches. In the current study, the electrodes were placed by the same experienced researcher according to the previous recommendations, and repeated measurements were conducted for each participant, which may be helpful in reducing the effects of crosstalk.

It should be noted that a majority of subjects in the current study have also participated in another experiment, the results of which have been published by Wang et al. (2022). However, the previous research has conducted a comparison of muscle activation and concomitant intermuscular coupling of antagonist muscles among bench presses with three different instability degrees, while in the current study, the effect of fatigue on intermuscular EMG-EMG coupling during bench press exercise has mainly concerned. Although the two studies shared nearly the same research method, the experiments of the two study have been conducted separately and the research objectives were completely different.

## 5 Conclusion

In conclusion, significant decrease of BB-TB and increase of AD-TB intermuscular phase synchronization index in gamma frequency band were observed in the second half of 60% 1RM fatiguing bench press exercise compared to the first half contraction. The results indicated a decrease of interconnection between synchronized cortical neurons and the motoneuron pool of BB and TB, and an increase of interconnection between AD-TB muscles. The changes of intermuscular coupling may be related to the supraspinal modulations to compensate for the decrease of muscle force as well as a result of unbalanced changes of agonist and antagonist muscle contractility.

## Data Availability

The raw data supporting the conclusions of this article will be made available by the authors, without undue reservation.
